# Effects of Chinese medicinal herbs on expression of brain-derived Neurotrophic factor (BDNF) and its interaction with human breast cancer MDA-MB-231 cells and endothelial HUVECs

**DOI:** 10.1186/s12906-017-1909-7

**Published:** 2017-08-12

**Authors:** Jen-Hwey Chiu, Fang-Pey Chen, Yi-Fang Tsai, Man-Ting Lin, Ling-Ming Tseng, Yi-Ming Shyr

**Affiliations:** 1 Institute of Traditional Medicine, School of Medicine, National Yang-Ming University, Taipei, Taiwan Republic of China; 2 Comprehensive Breast Health Center & Division of General Surgery, Department of Surgery, Taipei Veterans General Hospital, Taipei, Taiwan Republic of China; 3 Division of General Surgery, Department of Surgery, Cheng-Hsin General Hospital, Taipei, Taiwan Republic of China; 4 Center of Traditional Medicine, Taipei Veterans General Hospital, Taipei, Taiwan Republic of China; 5 Institute of Clinical Medicine, School of Medicine, National Yang-Ming University, Taipei, Taiwan Republic of China; 6 Department of Surgery, Faculty of Medicine, School of Medicine, National Yang-Ming University, Taipei, Taiwan Republic of China

**Keywords:** BDNF, Breast cancer, Endothelial cell, Chinese herbal medicine

## Abstract

**Background:**

Our previous study demonstrated that an up-regulation of the Brain-Derived Neurotrophic Factor (BDNF) signaling pathway is involved the mechanism causing the recurrence of triple negative breast cancer. The aim of this study is to investigate the effects of commonly used Chinese medicinal herbs on MDA-MB-231 and HUVEC cells and how they interact with BDNF.

**Methods:**

Human TNBC MDA-MB-231 cells and human endothelial HUVEC cells were used to explore the effect of commonly used Chinese herbal medicines on cancer cells alone, on endothelial cells alone and on cancer cell/endothelial cell interactions; this was done via functional studies, including migration and invasion assays. Furthermore, Western blot analysis and real-time PCR investigations were also used to investigate migration signal transduction, invasion signal transduction, and angiogenic signal transduction in these systems. Finally, the effect of the Chinese medicinal herbs on cancer cell/endothelial cell interactions was assessed using co-culture and ELISA.

**Results:**

In terms of autoregulation, BDNF up-regulated TrkB gene expression in both MDA-MB-231 and HUVEC cells. Furthermore, BDNF enhanced migration by MDA-MB-231 cells via Rac, Cdc42 and MMP, while also increasing the migration of HUVEC cells via MMP and COX-2 expression. As measured by ELISA, the Chinese herbal medicinal herbs *A. membranaceus, P. lactiflora, L. chuanxiong, P. suffruticosa* and *L. lucidum* increased BDNF secretion by MDA-MB-231 cells. Similarly, using a co-culture system with MDA-MB-231 cells, *A. membranaceus* and *L. lucidum* modulated BDNF-TrkB signaling by HUVEC cells.

**Conclusion:**

We conclude that BDNF plays an important role in the metastatic interaction between MDA-MB-231 and HUVEC cells. Some Chinese medicinal herbs are able to enhance the BDNF-related metastatic potential of the interaction between cancer cells and endothelial cells. These findings provide important information that should help with the development of integrated medical therapies for breast cancer patients.

**Electronic supplementary material:**

The online version of this article (doi:10.1186/s12906-017-1909-7) contains supplementary material, which is available to authorized users.

## Background

Breast cancer is a common female cancer worldwide and is also common in Taiwan [1, 2]. Triple negative breast cancer (TNBC), which comprises 15% of all breast cancers, is characterized by its early occurrence in younger women and its aggressive behavior, including a high recurrence rate and distant metastasis, Owing to a lack of specific receptors, TNBC shows strong resistance to chemotherapy, hormone therapy and targeted therapy [3]. Although many biomarkers associated specifically with various subtypes of TNBC have been identified [4] and several targeted therapies, such as luminal androgen receptor (LAR) and tyrosin kinase inhibitors, have been investigated in clinical trials [5, 6], long-term outcomes indicate that they seem to have limit usefulness when treating TNBC patients [7].

Brain-derived neurotrophic factor (BDNF) is a member of the “neutrophin” family. This protein supports the survival of existing neurons, encourages the growth of new neurons, helps with the creation of new synapses [8] and is involved in neural development/regeneration, muscle repair/regeneration, and differentiation [9]. Previously, BDNF and its related receptor signaling system (BDNF/TrkB) have been shown to play important roles in the regulation of cell proliferation and metastasis across many types of cancer, including colon cancer, lung cancer and cancer of the brain [10, 11]. In addition, BDNF is found to increase cell viability and is associated with a reduction of the apoptosis of breast cancer [12]. The expression of BDNF mRNA has been shown to correlate with increased nodal positivity and the local recurrence rate; furthermore, treatments with anti-BDNF and anti-TrkB-T1 antibodies result in tumor growth inhibition in tumor-bearing mice, which suggests that that this protein can be used as a marker to predict adverse pathological and clinical outcomes related to breast cancers [13]. Although controversy still exits as to the exact role of BDNF in tumor suppression and promotion, information concerning BDNF-related cancer-endothelial cell interactions are lacking [14].

There is an increasing trend whereby patients with breast cancer seek integrative medicine in order to relieve their discomfort during the conventional modern medical therapies used to treat their disease; these symptoms include hot flushes, cancer-related fatigue and insomnia [15]. The prevalence of the use of integrative therapy among women with breast cancer is around 75% for patients in non-Asian areas [16], and around 36% to 40% for Taiwanese patients [17], although these figures are likely to be underestimates. Unfortunately, many patients with breast cancer who use integrative therapies are not aware of the potential adverse effects of these additional treatments. For examples, the consumption of many Chinese herbal extracts can have an up-regulatory effect on the gene expression levels of ER and HER2 in vitro [18] as well as interacting with the effects of tamoxifen in vivo [19].

Our previous study targeting the molecular mechanisms that occur in paired tumor specimens obtained from patients with and without recurrence, have suggested that the BDNF signaling pathway is involved in the up-regulation of genes that are present in stage II and stage IIIA recurrent breast cancer [20]. Since cancer metastasis is highly related to tumor recurrence in TNBC, further investigation of the effects of Chinese medicinal herbals on BDNF-related metastatic potential, specifically on the interaction between cancer cells and endothelial cells, is likely to provide important information for TNBC patients when they have undertaken integrative therapies to alleviate their discomfort. Accordingly, the aim of this study was to investigate the effects of Chinese medicinal herbs on the BDNF-related interaction between MDA-MB-231 and HUVEC cells.

## Methods

### Cell line and reagents

The human triple negative breast cancer cell line MDA-MB-231 (ER-low, PR-low and HER2-low) was obtained from the American Type Culture Collection (ATCC, Manassas, VA, USA) and was maintained in F12 MEM (NO.12400–024,Gibco, NY,USA) supplemented with 10% FBS, 2 mM L-glutamine and penicillin/streptomycin and cultured at 37 °C in a humidified atmosphere containing 5% CO_2_. The human umbilical vein endothelial cell line (HUVECs) (BCRC No.H-UV001) was obtained from the Taiwan Medical Cell and Microbial Resources, Food Industry Research Development Institute, Taiwan, and was cultured in Medium 200 supplemented with low serum growth supplement (Invitrogen, Carlsbad, CA, USA). Cells at passages from 3 to 10 were used for all experiments. Recombinant human BDNF (PeproTech, Rocky Hill, NJ, USA) was used as a positive control and was purchased commercially.

### Preparation of Chinese herbal extracts

**Fig. 1:**
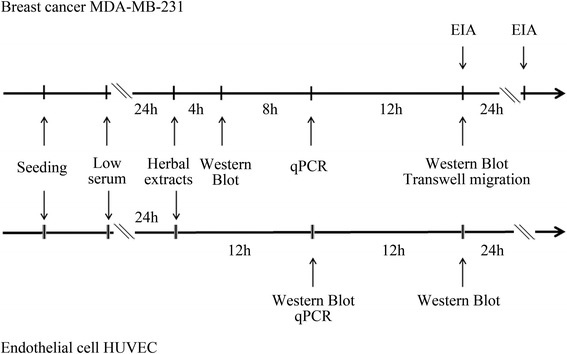
Experimental protocols. Two systems were used, namely, a triple negative MDA-MB-231 breast cancer cell line and a human umbilical endothelial cell line (HUVEC), for the study of autocrine and paracrine BDNF-TrkB loop regulation

Nine Chinese single herbs were used in this study. They were *Scutellariae baicalensis, Astragulus membranaceous, Salviae miltiorrhizae, Angelica sinensis, Paeonia lactiflora, Ligusticum chuanxiong, Anemarrhenae rhizome, Paeonia suffruticosa,* and *Ligustrum lucidum*. Chinese herbal extracts of these plants, denoted here as CHEs, were obtained from a GMP company (Sun Ten Pharmaceutical Co, Ltd., Taiwan) and had been authenticated by Ministry of Health and Welfare, Taiwan. The voucher specimens were prepared as water extracts of the CHEs by the company, which followed the standard GMP procedures in order to obtain compositions similar to those used clinically. The contents of the CHEs were standardized using high performance liquid chromatography (Additional file 1) [18]. To decrease any confounding effect caused by the presence of lipopolysaccharide contamination during the herbal preparation, polymyxin B (10 μg/mL) was administration routinely during each experiment. The protocol for the herbal treatment of the MDA-MB-231 and HUVEC cell lines and the evaluation parameters are shown in Fig. 1.

### Measurement of BDNF levels

The level of BDNF in the culture medium was evaluated by ELISA using a commercially available protocol (Quantikine ELISA Human BDNF). In brief, MDA-MB-231 (4 X10^5^/well in a six-well plate) cells were cultured for 1 day, which was followed by washing with PBS and the addition of low serum F-12 DMEM (0.1% FBS) with/without herbal extract treatment for 24 h and 48 h. Next the cultured medium or the conditioned medium was collected and centrifuged (300 x g) for 5 min. The level of BDNF in the supernatant from the medium was then measured by ELISA, and normalized against the cell number for CHE treatment.

### Cell migration assay

In vitro cell migration assays were carried out on either the MDA-MB-231 cells or the HUVEC cells. These were performed using a cell culture insert (NO.80209, ibidi, Munich, Germany*)*. In brief, 2 × 10^4^ cells were seeded into the insert on a 3.5 cm Petri dish overnight, which was followed by low serum (1% FBS) starvation for 24 h. Following this, the cells were washed with PBS and the inserts removed. Next the cells were allowed to continue to be cultured either with or without herbal extract treatment. After a 24 h-incubation, the migrating cells were examined under a light microscope and photographed. The percentage of migratory cells (or the area) was calculated by comparing the treated group with the vehicle group. Neutralizing antibody against BDNF (GTX 16327, GeneTex, Inc., San Antonio, Texas, USA), non-specific antibody regarding BDNF (normal rabbit serum) and TrkB receptor inhibitor (GNF5837, MedChem Express, NJ, USA) were used to inhibit the BDNF-related migration activity associated with the MDA-MB-231 culture conditioned medium.

### Transwell co-culture assays to assess the chemotaxic interaction between cancer cells and endothelial cells

MDA-MB-231 cells with a density of 8 × 10^4^ cells/well were cultured in the upper chamber (for the invasion assay) or lower chamber (for the chemotaxic interaction assay) of a Transwell system (ThinCertTM cell culture inserts, 24 well, 8 μm, Greiner bio-one, Switzerland) for 1 d and this was followed by replacement of the culture medium with low serum (0.1% FBS) F-12 DMEM with/without each of the Chinese herbal extracts (1 μg / mL) for another day. Next HUVECs with a density of 5 × 10^4^ cells/well were cultured in the upper chamber (for the chemotaxic interaction assay), which was placed onto the above-mentioned pretreated lower chamber. For invasion assay, MDA-MB-231 cells were cultured in the upper chamber with a reduced concentration of matrix gel (50 μg / mL/well). After 9 h of co-culture, the cells on the reverse side of upper chamber membrane were fixed and stained with 2% crystal violet for 10 min, then washed and photographed. The migrating cells were examined under a light microscope and photographed. The percentage of migrating or invasion cells was calculated by comparing the experimental group against the vehicle group.

### Western blotting analysis

Cultured MDA-MB-231 and HUVECs were lysed in a buffer containing 150 mM KCl, 10 mM Tris pH 7.4, 1% Triton X-100, phosphatase inhibitor and protease inhibitors cocktail (Complete Mini; Roche, Mannheim, Germany). The protein concentration of each cell homogenate was then measured using the Bradford’s method [21]. Next 30 μg of each protein sample was loaded onto a gel and the proteins separated by 10% SDS-PAGE, after which the separated proteins were transferred onto a nitrocellulose membrane (Hybond-C; Amersham Biosciences, NJ, USA). The membrane was then blocked with 5% bovine serum albumin, which was followed by probing with a series of specific primary antibodies against VEGFA (Genetex, GTX 102643), TrkB (BD, 610,101), BDNF (Genetex, GTX62495), COX-2(Cayman 160,112), MMP2 (Cell Signaling, #13132), MMP9 (Cell Signaling, #13667), p-Rac1/cdc42 Ser71 (Cell Signaling #2461), Rac1/2/3 (Cell Signaling, #2465), cdc42 (Cell Signaling, #2466), and RhoA (Cell Signaling #2117).

### Total RNA extraction and reverse transcription-PCR

Total RNA was isolated by a modified version of the single-step guanidinium thiocyanate method [22] (TRI REAGENT, T-9424, Sigma Chem. Co., St. Louis, MO, USA). Complementary DNA (cDNA) was prepared from the total RNA using a First Strand cDNA Synthesis Kit (Invitrogen, CA, USA). The level of de novo gene synthesis of each experimental group was measured by reverse transcriptase-polymerase chain reaction (RT-PCR). The primers pairs used and the annealing temperature were BDNF (Forward 5′-TGG CTG ACA CTT TCG AAC AC-3′; Reverse 5′-CCT CAT GGA CAT GTT TGC AG-3′; 51.8 °C), TrkB (Forward 5′-AGG GCA ACC CGC CCA CGG AA-3′; Reverse 5′-GGA TCG GTC TGG GGA AAA GG-3′; 56 °C), COX2 (Forward 5′-GCT GAG CCA TAC AGC AAA TCC-3′; Reverse 5-’ GGG AGT CGG GCA ATC ATC AG- 3′; 55 °C), MMP2 (Forward 5′-GCT GGC TGC CTT AGA ACC TTT C-3′; Reverse 5′-GAA CCA TCA CTA TGT GGG CTG AGA-3′; 57 °C), MMP9 (Forward 5′-GCA CGA CGT CTT CCA GTA CC-3′; Reverse 5′-GCA CTG CAG GAT GTC ATA GGT-3′; 55 °C), MMP13 (Forward 5′-GAC TTC CCA GGA ATT GGT GA-3′; Reverse 5′-TGA CGC GAA CAA TAC GGT TA-3′; 50 °C), eNOS (Forward 5′-CCC TTC AGT GGC TGG TAC AT-3′; Reverse5’-TAT CCA GGT CCA TGC AGA CA-3′; 52 °C), VEGFA (Forward 5′-CTT GCC TTG CTG CTC TAC C-3′; Reverse 5′-CAC ACA GGA TGG CTT GAA G-3′; 55 °C) and VEGFR2 (Forward 5′-GTG ACC AAC ATG GAG TCG TG-3′; Reverse 5′-CCA GAG ATT CCA TGC CAC TT-3′; 55 °C), all of which were obtained commercially. Any possible contamination of the PCR components was excluded by performing a PCR reaction with these components in the absence of the RT product (non-template control, NTC) and this was carried out for each set of experiments. Quantification of the RNA transcripts were expressed as mean + SEM by relating the mRNA data to the same data for various housekeeping genes using the formula A (target gene)/B (endogenous gene) = 2 ^–∆CT^. All samples were analyzed using either three or four independent experiments.

### Statistics

Data are expressed as the mean ± SEM. Differences between groups at a given time/dose point were identified by one-way ANOVA, which was followed by Dunnet’s post hoc test. Statistical comparisons between two independent groups were determined by the Mann-Whitney U test or the Student’s t test as appropriate. A *p* value of <0.05 was considered statistically significant compared to the vehicle or the no treatment group.

## Results

**Fig. 2:**
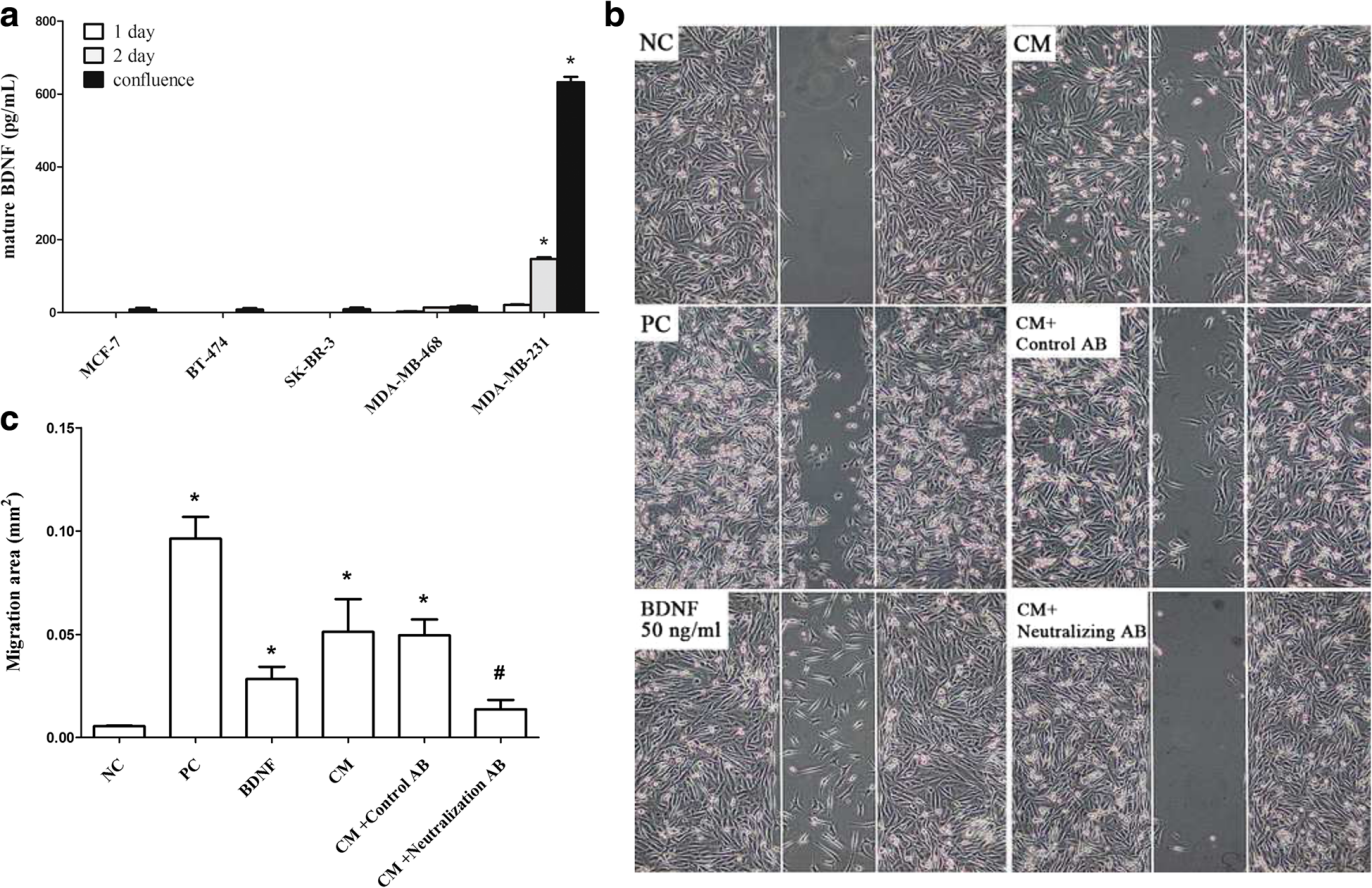
BDNF levels in various culture media and the protein’s effect on cell migration using the MDA-MB-231 line. Different breast cancer cell lines, such as MCF-7, BT-474, SK-BR-3, MDA-MB-468 and MDA-MB-231 (4 × 10^5^/well), were cultured for 1 day and this was followed by replacement by low serum medium at 24 h (white bar), 48 h (grey bar) and confluence (black bar). Next the culture media were collected to allow the BDNF level in each medium (**a**) to be measured by ELISA as described in Methods. A cell migration assay (**b**) using a cell culture insert, as described in Methods, was carried out in the presence of serum free medium (the negative control, NC), 10% FBS medium (positive control, PC) BDNF (50 ng/mL), or 2-day-cultured medium (conditioned) medium (CM) using MDA-MB-231 cells without or with pretreatment with a neutralization antibody against BDNF or non-specific antibodies. The percentage of migratory cells (**c**) was measured by comparing the treated group with the vehicle group. Data are expressed as mean + SEM. *, *p* < 0.05 compared to fresh prepared medium (day 0) or serum free (negative control), one way ANOVA. #, *p* < 0.05 compared to CM-treated group (n = three or four independent experiments in each group) by Mann-Whitney U test

We measured the BDNF level in the cultured media of various different breast cancer cell lines and the results showed that the MDA-MB-231 cells, but not the various other cell types, secrete BDNF into the culture media in a time-dependent manner (Fig. 2a). When analyzed by migration assay, the increase in migratory activity induced by administration of the 2-day-culture medium obtained from MDA-MB-231 cells was able to be blocked by pretreatment with neutralization antibody against BDNF, but not by non-specific antibody (Fig. 2b, c). The results therefore suggest that BDNF promotes cell migration by the MDA-MB-231 cell line.

**Fig. 3:**
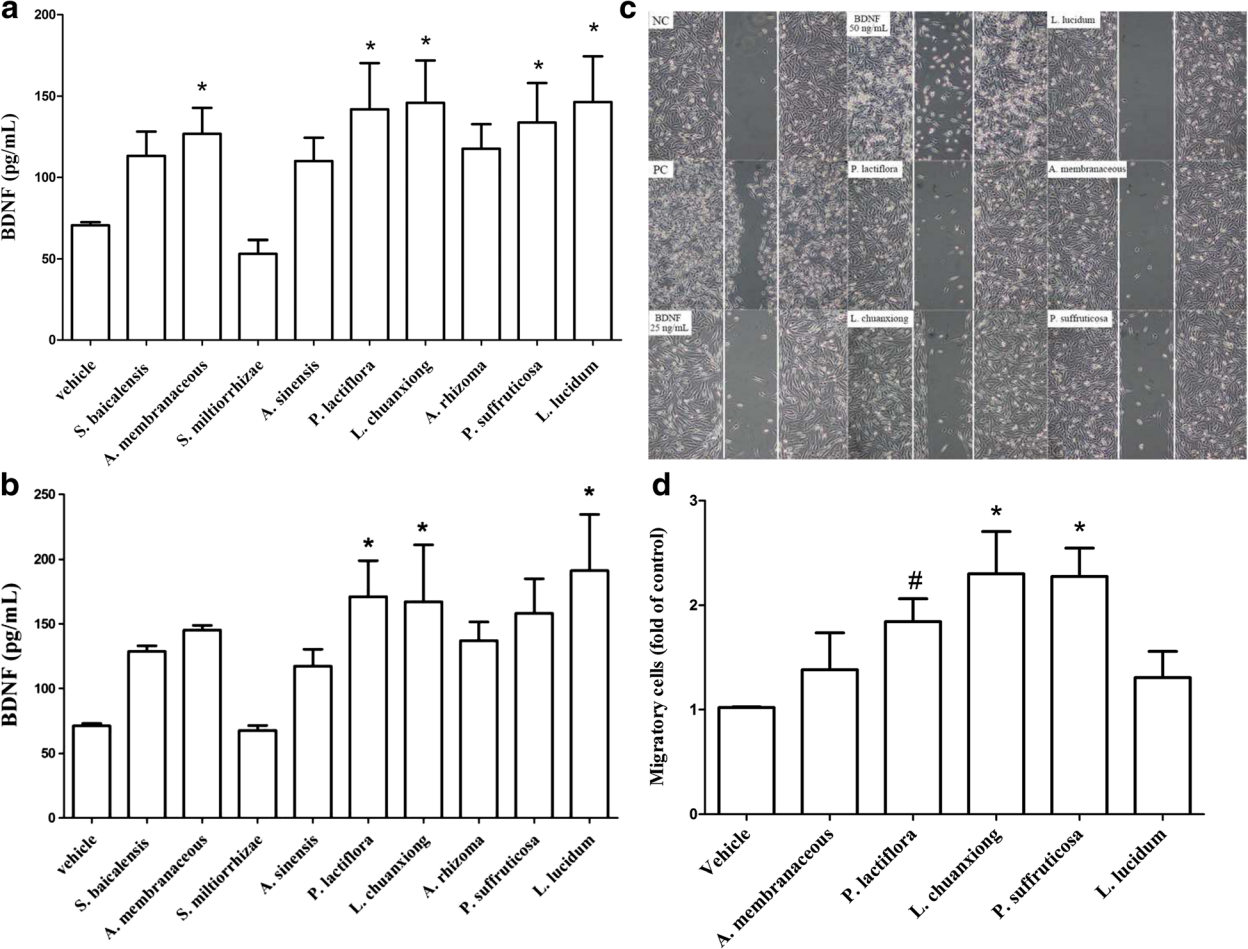
Effects of the various Chinese herbal extracts (CHE) on BDNF secretion, cell migration and cell invasion using the MDA-MB-231 line. MDA-MB-231 (4 × 10^5^/well) cells were cultured with low serum medium and this was followed by administration of each of the various CHEs individually (1 μg/mL for each herbal extract) for 24 h. Each culture medium was collected to allow the BDNF level (**a**) and BDNF level normalized against the cell number (**b**) to be measured by ELISA as described in Methods. CHE-induced cell migration (**c**) was evaluated by migration assay. Those herbs with migration-stimulating potential were also evaluated by invasion assay (**d**). The data are expressed as mean + SEM. *, *p* < 0.05 compared to vehicle group by repeatedly measured one way ANOVA (n = five independent experiments in each group)

To investigate the effects of Chinese herbal extracts on BDNF secretion, nine common CHEs were administrated to MDA-MB-231 cells for 24 h and the BDNF level in culture media were measured by ELISA. The results showed that *A. membranaceous, P. lactiflora, L. chuanxiong, P. suffruticosa,* and *L. lucidum* extracts were able to significantly increase BDNF secretion (Fig. 3a). If the measurements are normalized against the cell number for CHE treatment, only *P. lactiflora, L. chuanxiong,* and *L. lucidum* extracts show a significant increase in BDNF secretion (Fig. 3b) as well as a stimulation of the migratory activity (Fig. 3c) of the MDA-MB-231 cell line. Among these herbs, *P. lactiflora, L. chuanxiong,* and *P. suffruticosa* also showed an increase in the invasive activity (Fig. 3d), which suggests that these herbal extracts have the ability to promote the metastatic potential of the cancer cells.

**Fig. 4:**
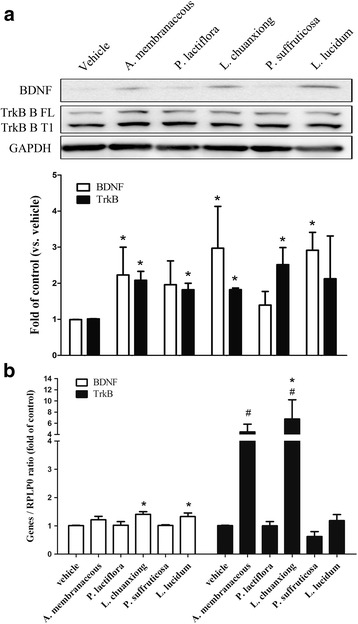
Effects of the various Chinese herbal extracts (CHE) on the gene expression levels of the BDNF autoregulation loop using MDA-MB-231 line. Cultured MDA-MB-231 cells without/with each CHE individual (1 μg/mL for each herbal extract) pretreatment were lysed and the gene expression levels of BDNF and TrkB at both the protein and mRNA level were analyzed by Western blot (**a**) and real-time PCR (**b**), respectively. Data are expressed as mean + SEM. *, *p* < 0.05 compared to vehicle group by Mann-Whitney U test (n = four or five independent experiments in each group)

Next, we examined the effects of the CHEs on the BDNF autoregulation loop of the MDA-MB-231 cell line. To do this, the gene expression levels of BDNF and TrkB in the cultured cells were analyzed. The results show that *A. membranaceous, L. chuanxiong,* and *L. lucidum* were able to significantly increase the expression of the BDNF protein by the MDA-MB-231 cell line. Furthermore, *A. membranaceous, P. lactiflora, L. chuanxiong,* and *P. suffruticosa* were able to significantly increase the expression of TrkB protein by the MDA-MB-231 cell line (Fig. 4a). In addition to the above, *L. chuanxiong,* and *L. lucidum* were able to up-regulate the expression of *BDNF* mRNA, while *A. membranaceous, and L. chuanxiong* were able to up-regulate the expression of *NTF2 (*TrkB) mRNA by the same cell line (Fig. 4b). Finally, there was found to be a correlation between BDNF and TrkB protein levels and between BDNF and TrkB mRNA levels in *L. chuanxiong*-treated cells. These results suggest that *L. chuanxiong* is able to modulate regulation via the BDNF-TrkB autocrine loop.

**Fig. 5:**
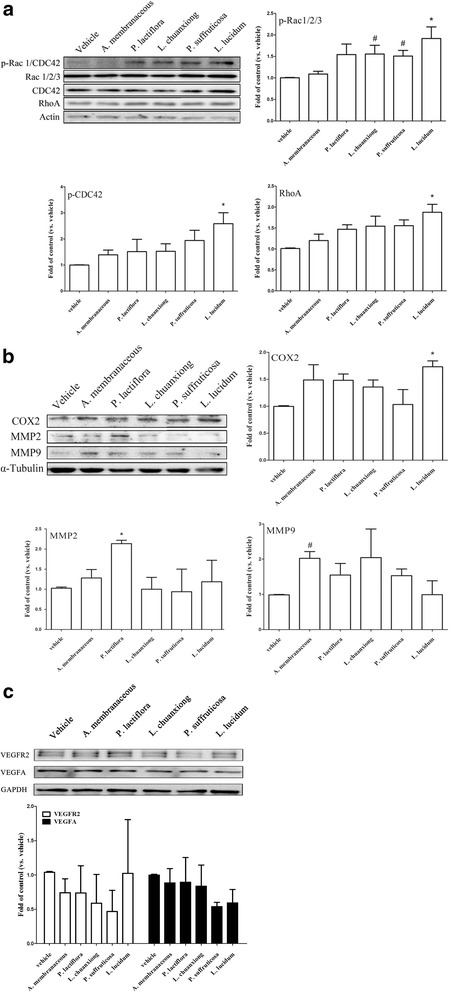
Effects of Chinese herbal extracts (CHE) on the expression levels of migratory proteins using MDA-MB-231 cells. Cultured MDA-MB-231 cells without/with a CHE (1 μg/mL for each herbal extract) pretreatment were lysed and the expression levels of various proteins related to migration, namely Rac 1/2/3, CDC42, Rho (**a**) and COX2, MMP2 and MMP9 (**b**) were analyzed by Western blot. A similar process was carried out for two angiogenesis related proteins, namely VEGFA and VEGFR2 (**c**) The results are expressed as mean + SEM. *, *p* < 0.05 compared to the vehicle group by repeatedly measured one way ANOVA; #, *p* < 0.05 by Student’s t test (n = three to five independent experiments in each group)

Next we investigated the effects of the CHEs on the expression of proteins that are involved in cell migration using MDA-MB-231 cells. The results showed that *L. chuanxiong, P. suffruticosa,* and *L. lucidum* increased p-Rac 1/2/3 protein expression (Fig. 5a), while *P. lactiflora* and *L. chuanxion* increased COX2 protein expression. Furthermore, *A. membranaceous and L. chuanxiong,* and *L. lucidum* significantly increased BDNF protein expression. Similarly, *P. lactiflora* increased MMP2 protein expression and *A. membranaceous* increased MMP9 protein expression (Fig. 5b). However, it should be noted that there was no significant change in the protein expression of various angiogenic factors, including VEGFA and VEGFR2, when the herb-treated cells were compared with the vehicle group (Fig. 5c). These results suggest that, while various herbs are able to induce cell migration and/or invasion, it would seem that none of the herbs are able to affect the angiogenic process. Taken together these findings suggest that there might be activation of a range of different signaling pathways by the individual herbal extracts.

**Fig. 6:**
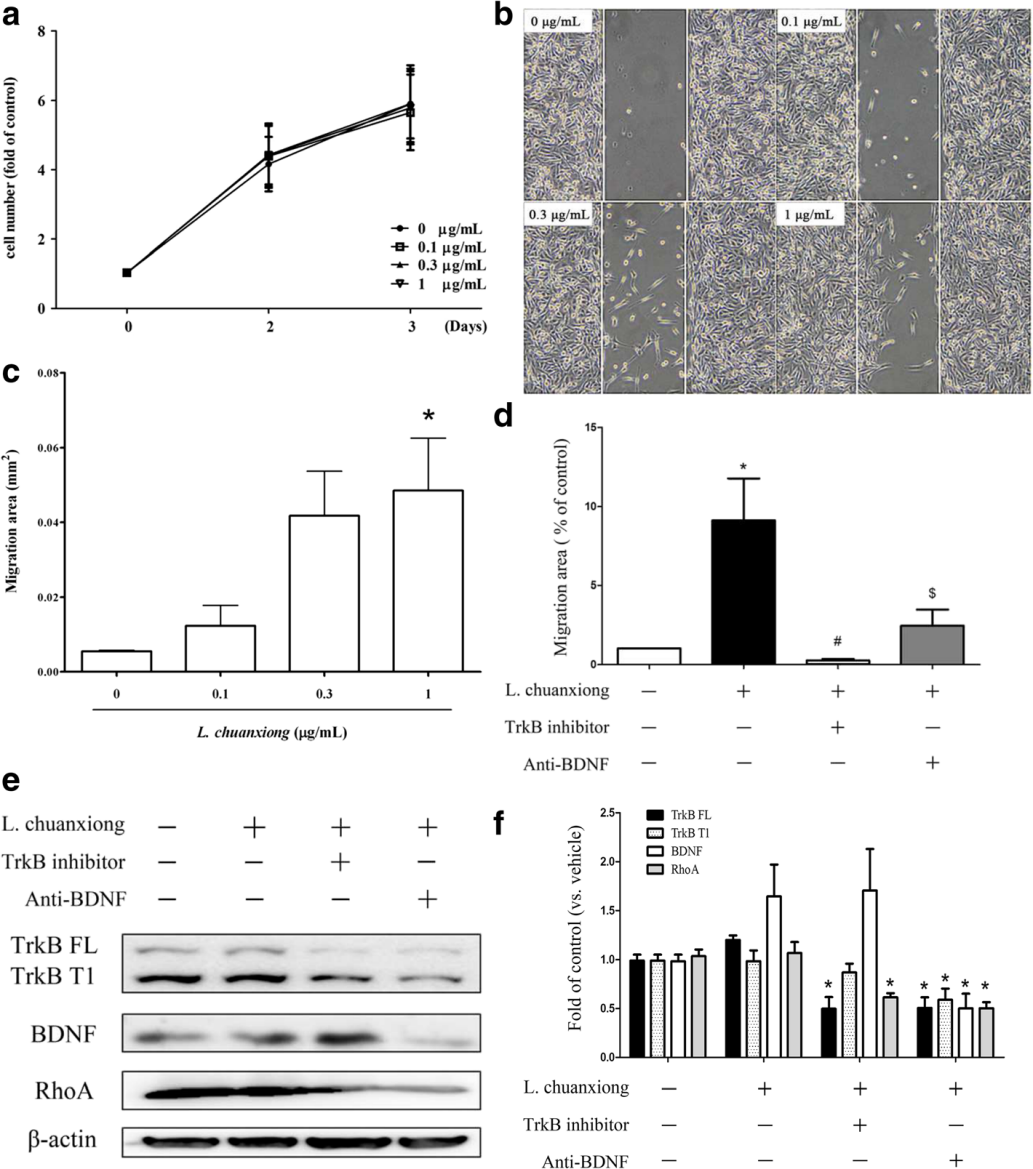
The role of BDNF-TrkB signaling in the *L. chuanxiong* induced responses within MDA-MB-231 cells. *L. chuanxiong*-treated MDA-MB-231 cells, without/with anti-BDNF antibody and with/without TrkB inhibitor pretreatment independently were evaluated by MTT assay (**a**) and migration assay (**b**, **c**, **d**). Furthermore, cells were lysed to allow the proteins present to be analyzed by Western blotting (**e**). The results were quantified (**f**) and are expressed as mean + SEM. *, *p* < 0.05 compared to vehicle group by repeatedly measured one way ANOVA; #, *p* < 0.05 by Mann Whitney test, $, < 0.05 by the Student’s t test (n = three to five independent experiments in each group)

To study in particular the role of BDNF-TrkB signaling in the context of the herb-induced responses produced by MDA-MB-231 cells, BDNF-neutralization antibody and TrkB inhibitor were used separately with *L. chuanxiong* extract to co-treat the MDA-MB-231 line. The results showed that *L. chuanxiong* (0.1-, 0.3-, 1 μg/mL) did not increase cell proliferation (Fig. 6a), but stimulated migratory activity (Fig. 6b), and that both anti-BDNF antibody and TrkB inhibitor, when used separately, were able to inhibit the induction of migratory activity by *L. chuanxiong* (Fig. 6c) using MDA-MB-231 cells. The *L. chuanxiong* induced increases in TrkB FL and BDNF expression were completely blocked by pretreatment with anti-BDNF antibody, while only TrkB, but not BDNF, expression was blocked by pretreatment with TrkB inhibitor (Fig. 6d, e). The results suggest that BDNF plays an important role in the *L. chuanxiong* induced BDNF-TrkB autocrine loop regulation found in MDA-MB-231 cells.

**Fig. 7:**
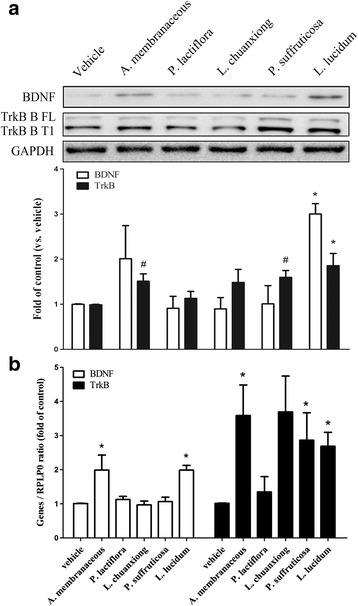
The effects of various Chinese herbal extracts (CHE) on the gene expression within the BDNF autoregulation loop using HUVECs. Cultured HUVEC cells with/without/with a given CHE (1 μg/mL for each herbal extract) pretreatment were lysed and the protein and mRNA gene expression levels of BDNF and TrkB were analyzed by Western blot (**a**) and real-time PCR (**b**), respectively. Data are expressed as mean + SEM. *, *p* < 0.05 compared to vehicle group by repeatedly measured one way ANOVA; #, *p* < 0.05 compared to vehicle group by Student’s t test (n = three to five independent experiments for each group)

Since BDNF in its mature form is secreted into the culture media of MDA-MB-231, this protein has the potential to affect adjacent cells associated with metastasis such as endothelial cells, thus there is a possibility of a paracrine effect. Therefore next we attempted to determine using HUVEC cells what was the effect of CHEs on the BDNF paracrinal loop. The results show that, in HUVEC cells, *L. lucidum* significantly increased the level of BDNF protein while *A. membranaceous, P. suffruticosa* and *L. lucidum* significantly increased the level of TrkB protein (Fig. 7a). Furthermore, *A. membranaceous* and *L. lucidum* up-regulated the expression level of *BDNF* mRNA, while *A. membranaceous, P. suffruticosa* and *L. lucidum* up-regulated the expression level of *NTF2 (*TrkB) mRNA using the same cell line (Fig. 7b). The results suggest that *A. membranaceous and L. lucidum* are able to modulate BDNF-TrkB autocrine loop regulation present in HUVEC cells

**Fig. 8:**
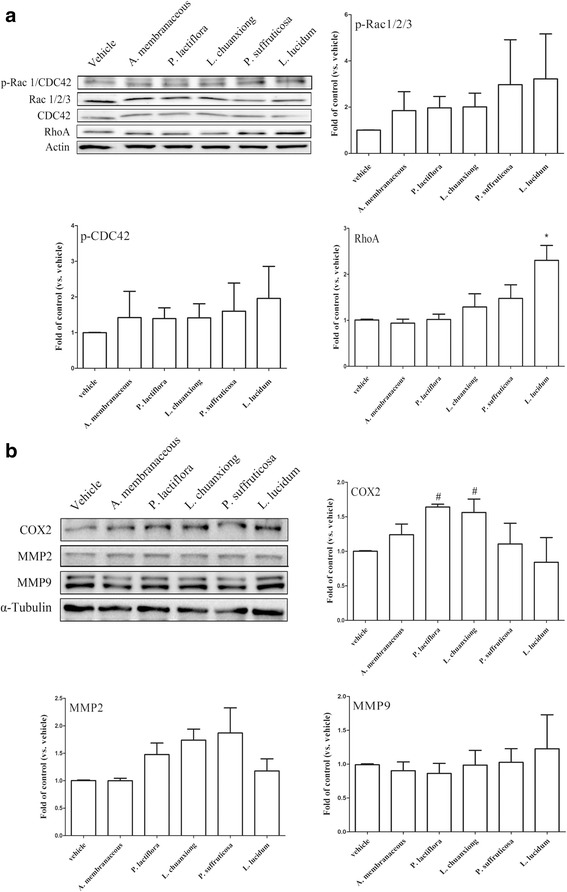
The effects of Chinese herbal extracts (CHE) on expression of protein involved in migration using HUVEC cells. Cultured HUVEC cells without/with CHE (1 μg/mL for each herbal extract) pretreatment were lysed and the expression levels of proteins related to migration, such as Rac 1/2/3, CDC42, Rho (**a**), COX2, MMP2 AND MMP9 (**b**), were analyzed by Western blotting. The results are expressed as mean *+* SEM. *, *p* < 0.05 compared to the vehicle group by repeatedly measured one way ANOVA; #, *p* < 0.05 compared to vehicle group by Mann-Whitney U test (n = three to five independent experiments for each group)

Finally, the effect of CHEs on migratory protein expression by HUVEC cells was analyzed by Western blotting. The results showed that, using HUVEC cells, *L. lucidum* increased Rho protein expression (Fig. 8a) while *P. lactiflora* and *L. chuanxiong* increased COX2 protein expression (Fig. 8b). These findings suggest that these proteins are likely to have the ability to stimulate the migratory ability of endothelial cells.

## Discussion

Cancer metastasis is a multistage process that requires interactions between cancer cells and non-malignant cells within the tumor microenvironment. In this article, the effect of the interaction between cancer cells (MDA-MB-231) and endothelial cells (HUVEC) on migratory potential was investigated. To our knowledge, we are the first to investigate the effect of Chinese herbal medicines on the BDNF-related metastatic potential of cancer-endothelial interactions. There is consensus that lipopolysaccharide contamination during herbal preparation might have an effect on the results of this type of experiment. To decrease such contamination effects, polymyxin B was administration routinely during each experiment [23]. The major limitation of this study is that only one dose of herbal extract was used. Furthermore, our previous study has shown that there was no cell cytotoxicity at the dose ranges used [18]. Nevertheless, further dose-related investigations on the herbs investigated in this study are mandatory, as well as being on going.

In this study, we found that BDNF affected not only MDA-MB-231 itself (an autocrine effect), but also affected HUVEC cells (a paracrine effect). The role of BDNF in the regulation of cell survival/growth has been described elsewhere regarding breast cancer [13], neuroblastoma and myeloma and these effects have been found to occur in both an autocrinal and a paracrinal manner [24, 25]. Accumulating evidence suggests that a reduction in the level of BDNF protein in specific areas of the brain provokes depressive-like behavior as well as affecting neurogenesis in vivo [26]. Recent investigations have demonstrated that some Chinese medicinal herbs have an antidepressant-like effect that occurs via an increase in the level of BDNF within the rat brain [27]. In addition, a compound herbal recipe (*Kai-Xin-San*), which is composed of Radix *Panax ginseng*, Rhizoma Smilacis Glabrae (*Fu-Ling*), Radix Polygalae (*Yuan-Zhi*) and Rhizoma Acori Tatarinowii (*Shi-Chang-Pu*), has been shown to increase BDNF expression and exerts a neuroprotective effect as well as an antidepressant-like effect via a synergistic mechanism that involves the multiple compounds present in this recipe [28].

For decades, there has been an increasing trend whereby patients with breast cancer seek integrative therapies that can include Chinese medicinal herbs with the aim of relieving their discomfort during cancer treatment. Although the role of BDNF in breast cancer remains controversial [14], it has become important to elucidate the effects of Chinese herbal medicines on BDNF production by cancer cells. In earlier studies, the secretion of MMPs has been proposed to make a significant contribution to the BDNF-induced cancer invasiveness of neuroblastoma and lung large cell neuroendocrine carcinoma [29, 30]. In addition to MMPs, cyclooxygenase-2 (COX-2) and prostaglandin E2 have also been shown to be involved in cancer development, angiogenesis and invasion. Previous investigations have also suggested that treatment with herbal extract can have an anticancer effect. One such example is Paeonol extract, which exerts an anticancer effect on colorectal cancer cells via an inhibition of PGE2 and COX-2 expression [31, 32]. There is consensus that the Rho A family and CDC42-Rac signaling, play independent roles in the various stages of actin reorganization, while COX2 and MMPs are involved in tumor invasion by many cancer types. Our findings indicate that the various herbal extracts are able to promote cell migration and/or invasion, which leads us to speculate that these herbal responses might occur via the activation of a number of distinct signaling pathways. It should be noted that there are corresponding changes in the levels of BDNF and TrkB at the protein level and BDNF and TrkB at the mRNA transcript level when cells are treated with *L. chuanxiong*, while there is a trend towards a correlation between these levels when cells are treated with *A. membranaceous*- and *L. lucidum*. The discrepancies found with the other herbal treatments may be due to the synergistic and/or antagonistic properties of the various different chemical compounds found in such herbal extracts. The complex mixture of chemicals present is very likely to induce complex mixture of drug-drug, cell-cell, and drug-cell interactions. In addition to the above, there are also a range of different responses when migratory protein expression levels were examined after treatment with the various herbal extracts when the MDA-MB-231 and HUVEC cell lines were compared. These cell lines have different embryonic origins and it is reasonable that there should be distinct and cell-specific responses to a given treatment when these different cell types are investigated.

There is consensus that angiogenesis plays an important role in cardioprotection. Recent studies have showed that an extract of *A. membranaceus* not only increases cell proliferation, migration and tube formation [33], but also that it has been found to affect nitric oxide production, a key regulator of angiogenesis, via the JAK2/STAT3 and ERK1/2 pathways in human endothelial HUVEC cells [34]. In addition to the above, there is evidence that BDNF induces the migration of endothelial cells via the BDNF-TrkB signaling pathway [35]. Our findings show that not only *L. lucidum,* but also *A. membranaceous*, is able to increase the level of TrkB protein in HUVEC cells and, furthermore, such treatment also up-regulate *BDNF* and *NTF2 (*TrkB) mRNA levels in the same cells, which might also explain the above-mentioned *A. membranaceous* effects.

## Conclusions

In summary, we have demonstrated that many Chinese medicinal herbs are able to enhance BDNF-TrkB signaling in both an autocrine and a paracrine manner. These findings provide important information that should help with the development of better integrative medical therapies that can be used by patients during the treatment of breast cancer.

## Additional file

Additional file 1: High performance of liquid chromatography for Chinese herbal extracts. (PDF 310 kb)

## Abbreviations

BDNF: Brain-Derived Neurotrophic Factor
CHE: Chinese herbal extracts
HUVEC: Human umbilical vein endothelial cell
MMPs: Matrix metalloproteinase
TNBC: Triple negative breast cancer
