# Supplementary material for: Effects of Chinese medicinal herbs on expression of brain-derived Neurotrophic factor (BDNF) and its interaction with human breast cancer MDA-MB-231 cells and endothelial HUVECs
Source: BMC Complement Altern Med. 2017 Aug 12;17:401. doi: 10.1186/s12906-017-1909-7 (PMC5554408; doi:10.1186/s12906-017-1909-7)

S. miltiorrhiza -Tanshinone II A (HPLC)

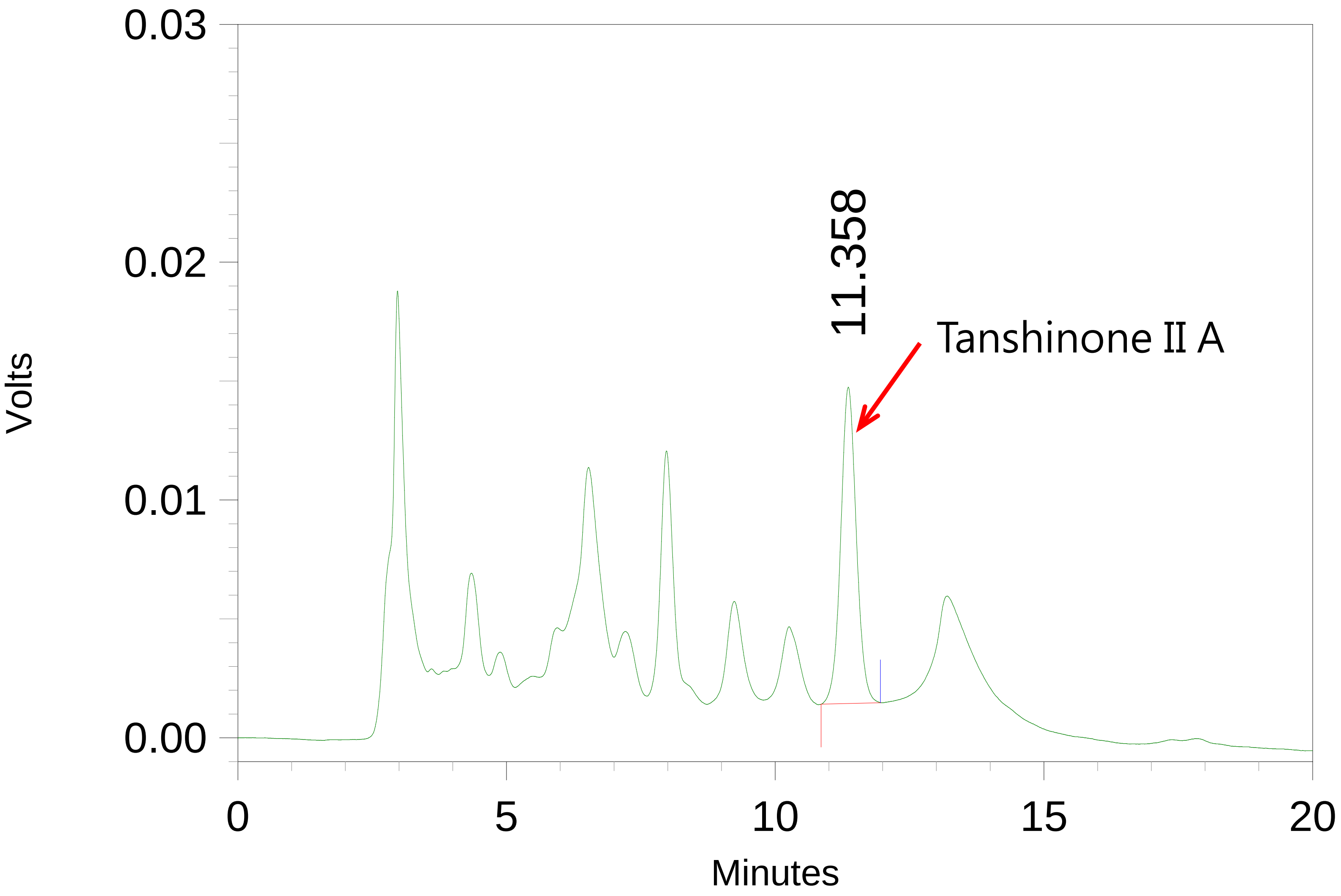

S. baicalensis -Baicalin (HPLC)

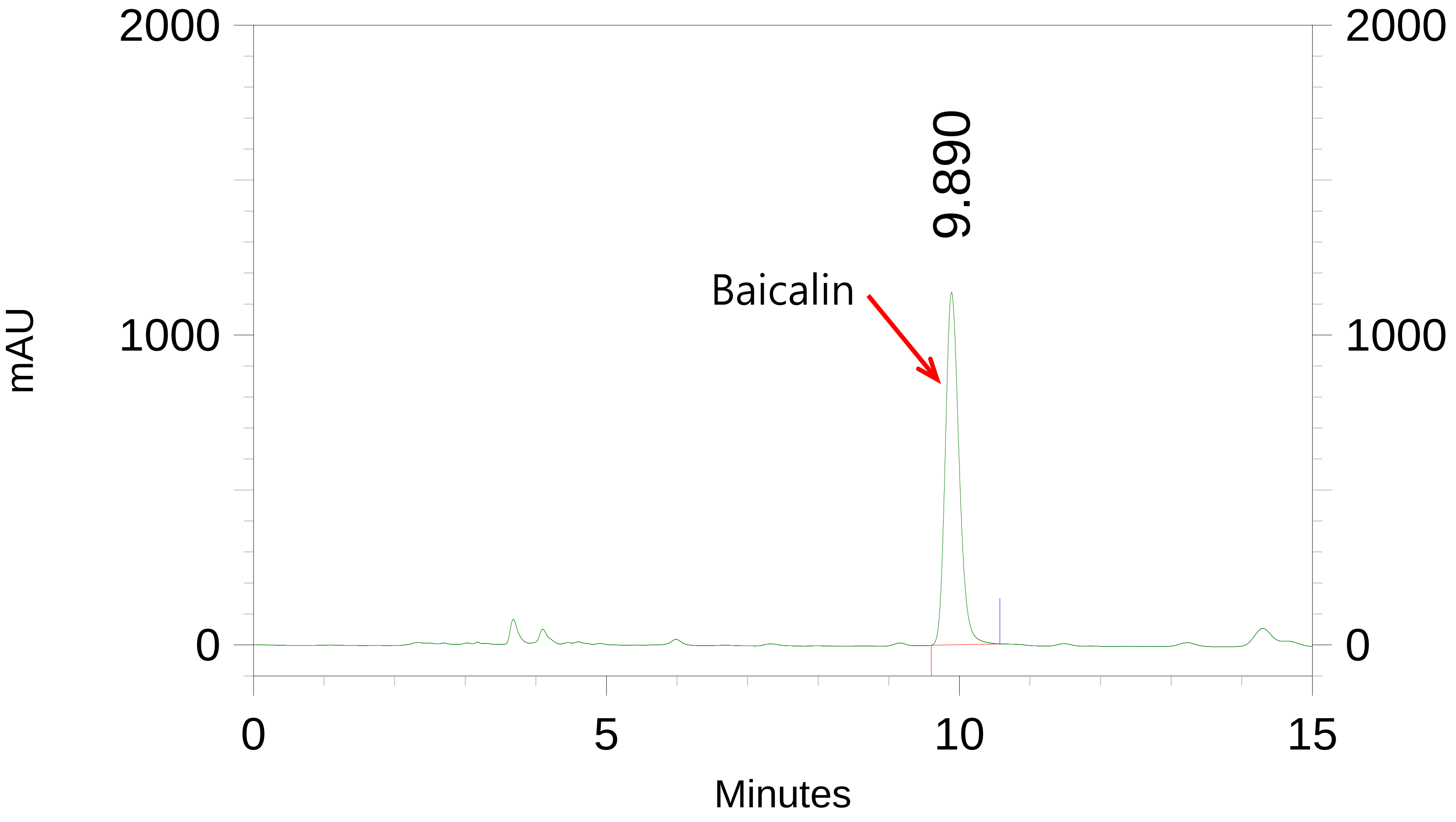

P. lactiflora -Paeoniflorin (HPLC)

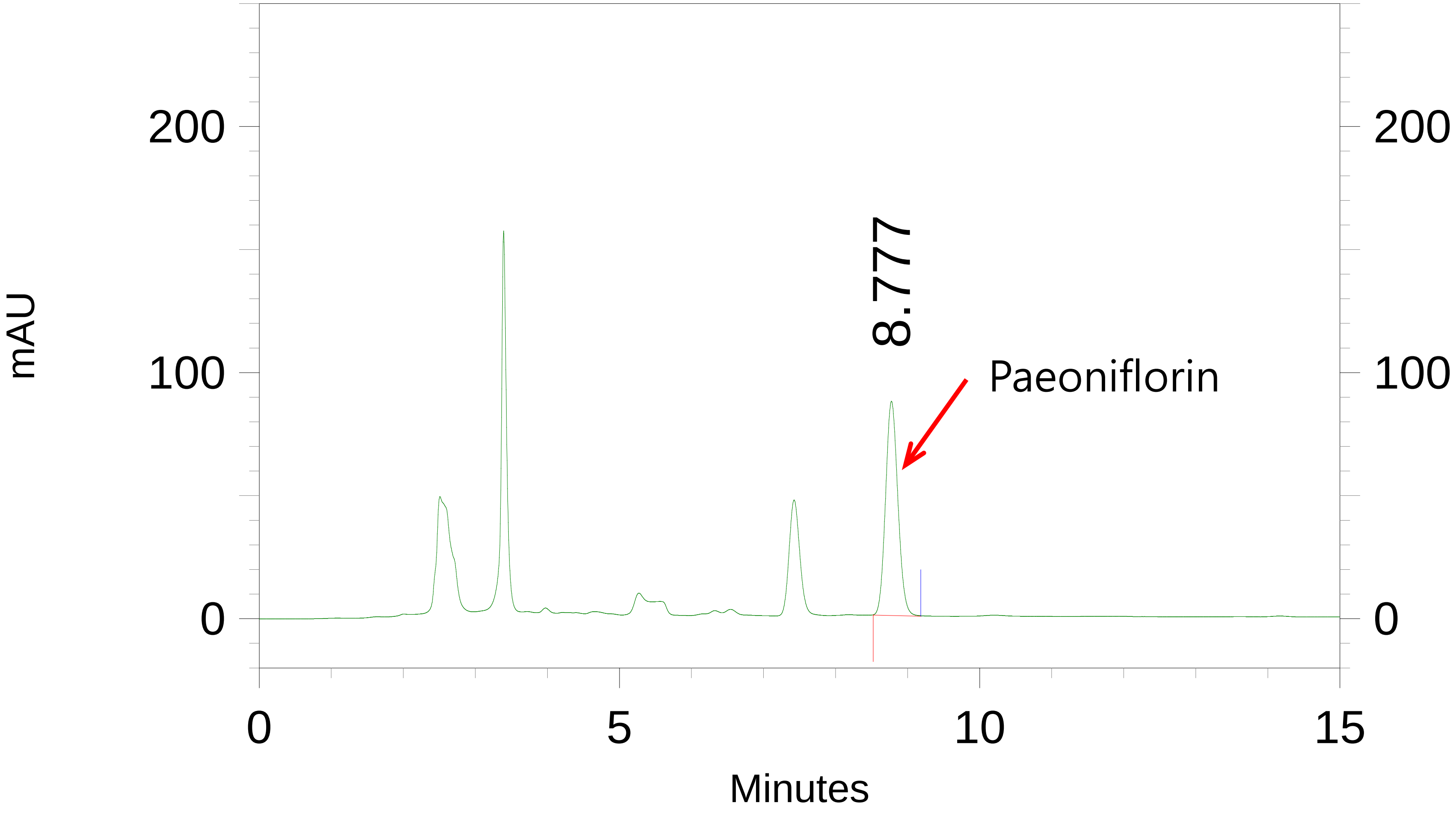

P. suffruticosa -Paeoniflorin (HPLC)

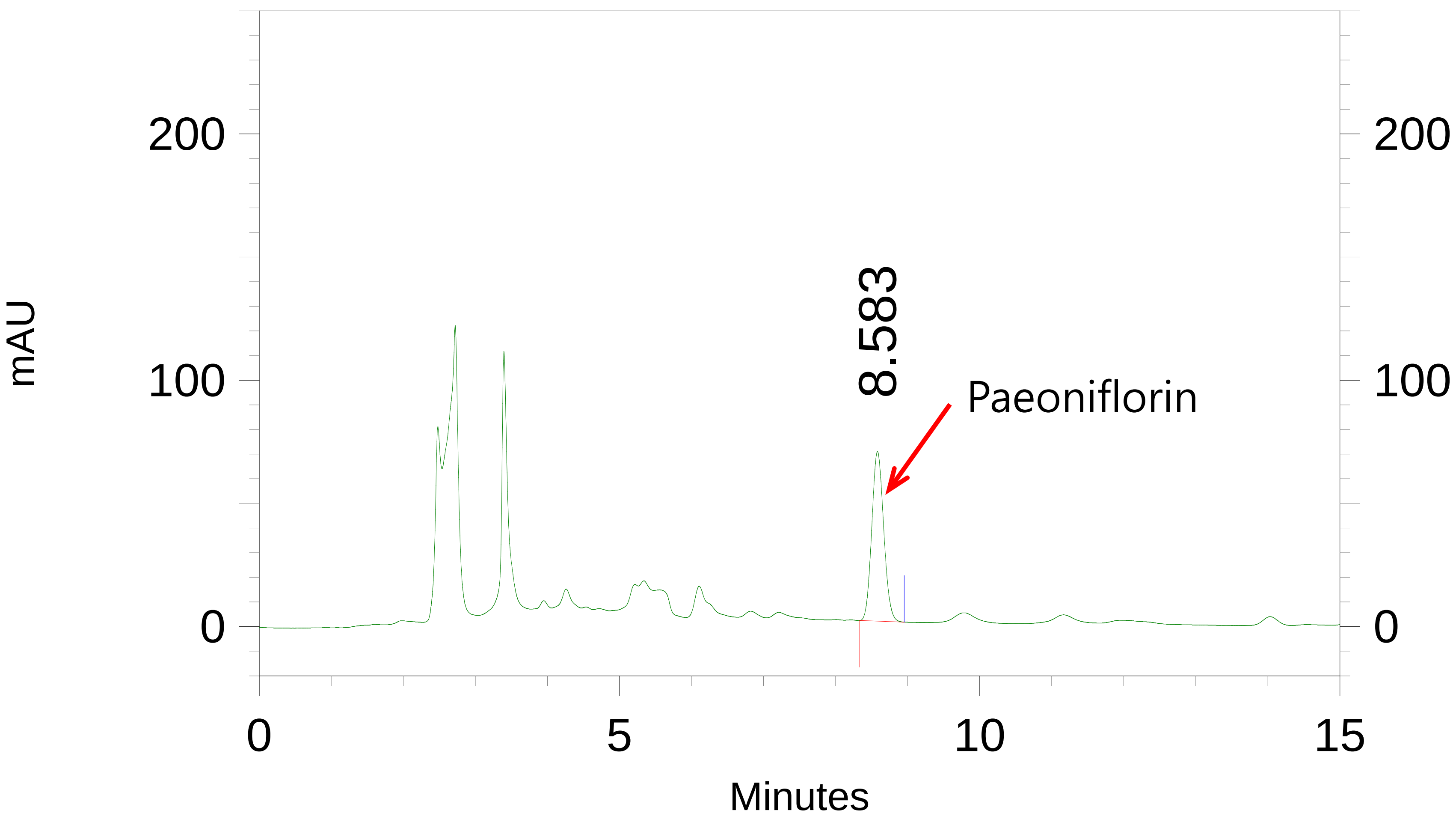

P. suffruticosa -Paeonol (HPLC)

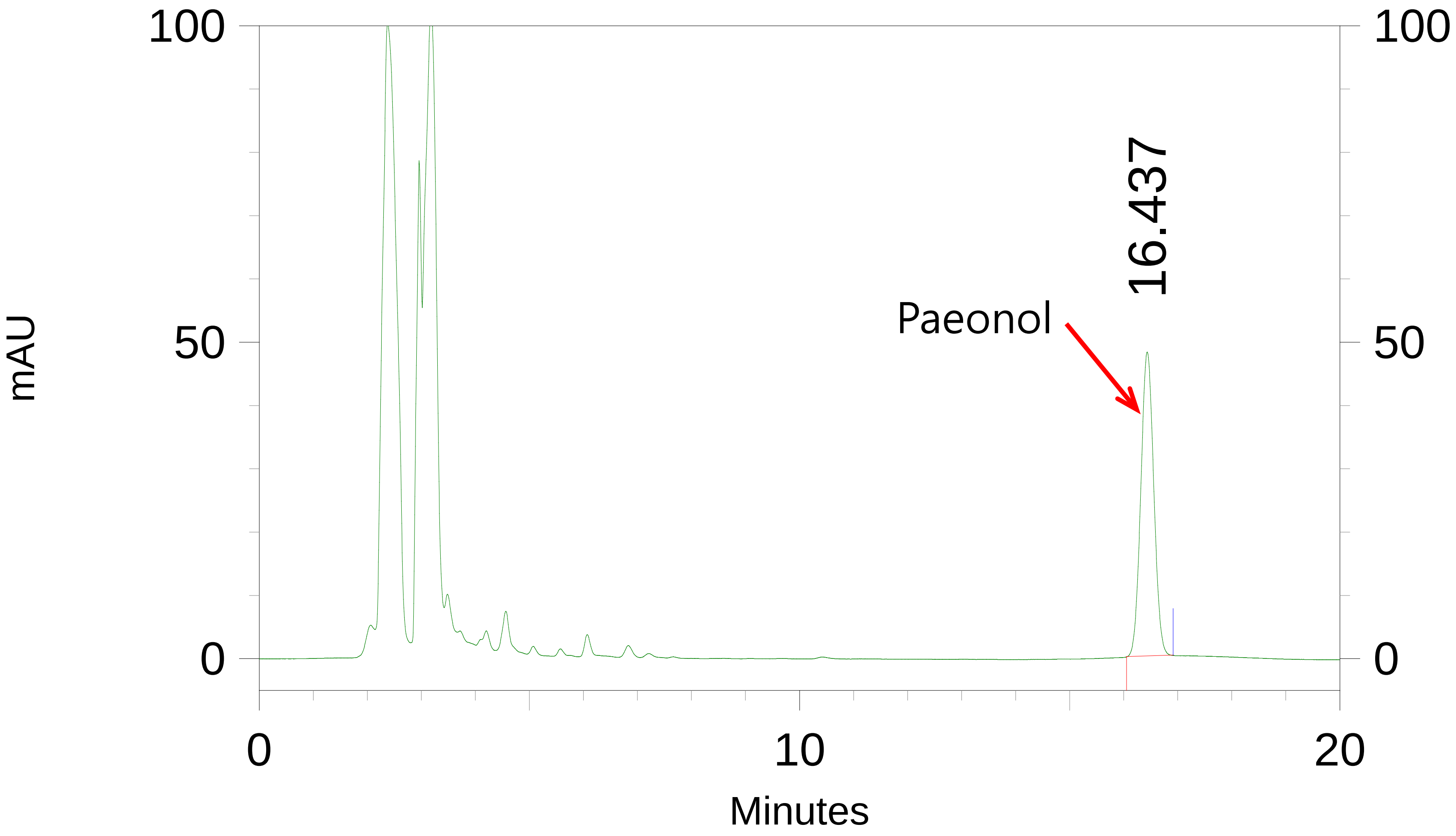

L. chuanxiong -Ferulic acid (HPLC)

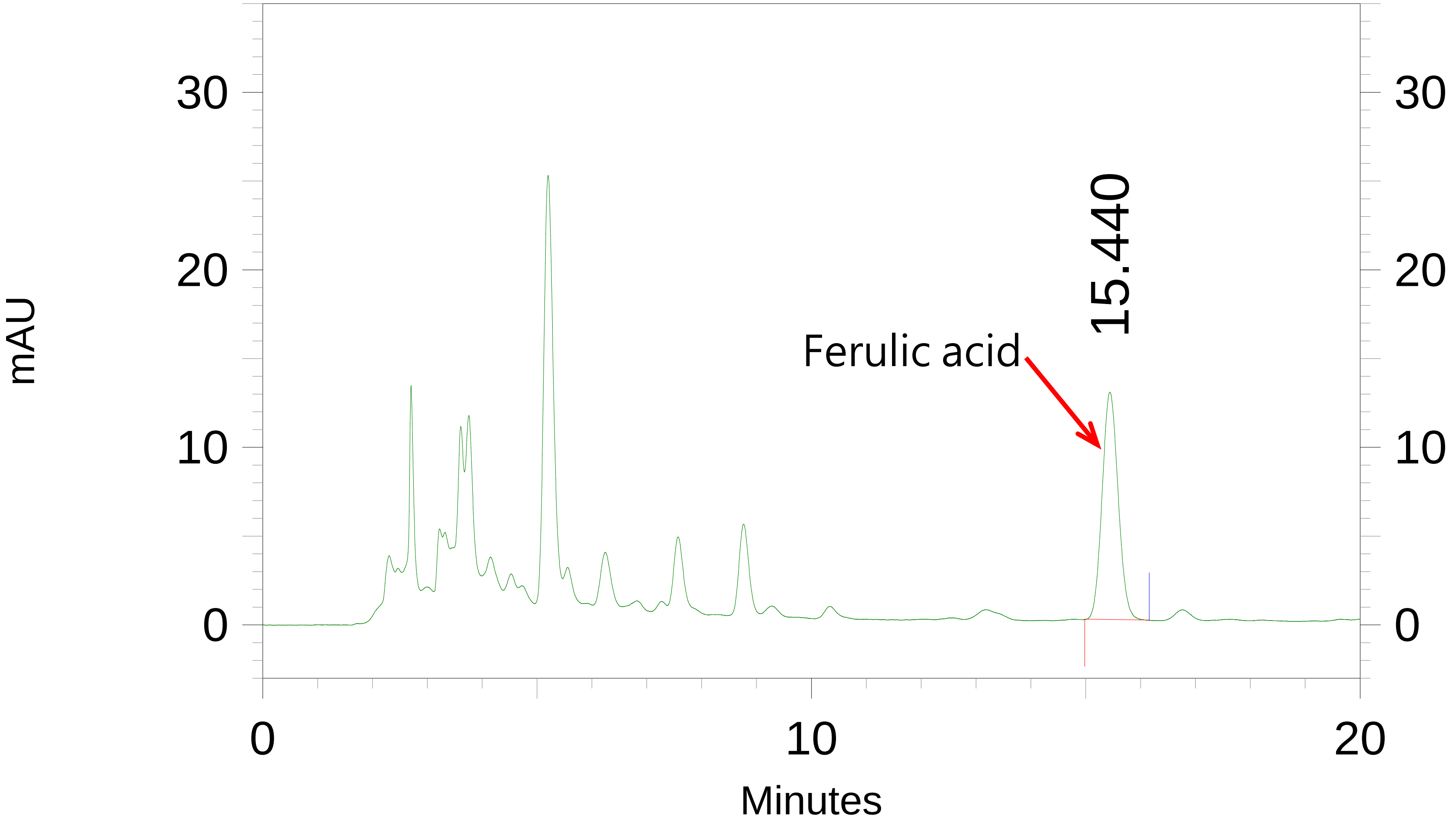

A. rhizoma -Mangiferin (HPLC)

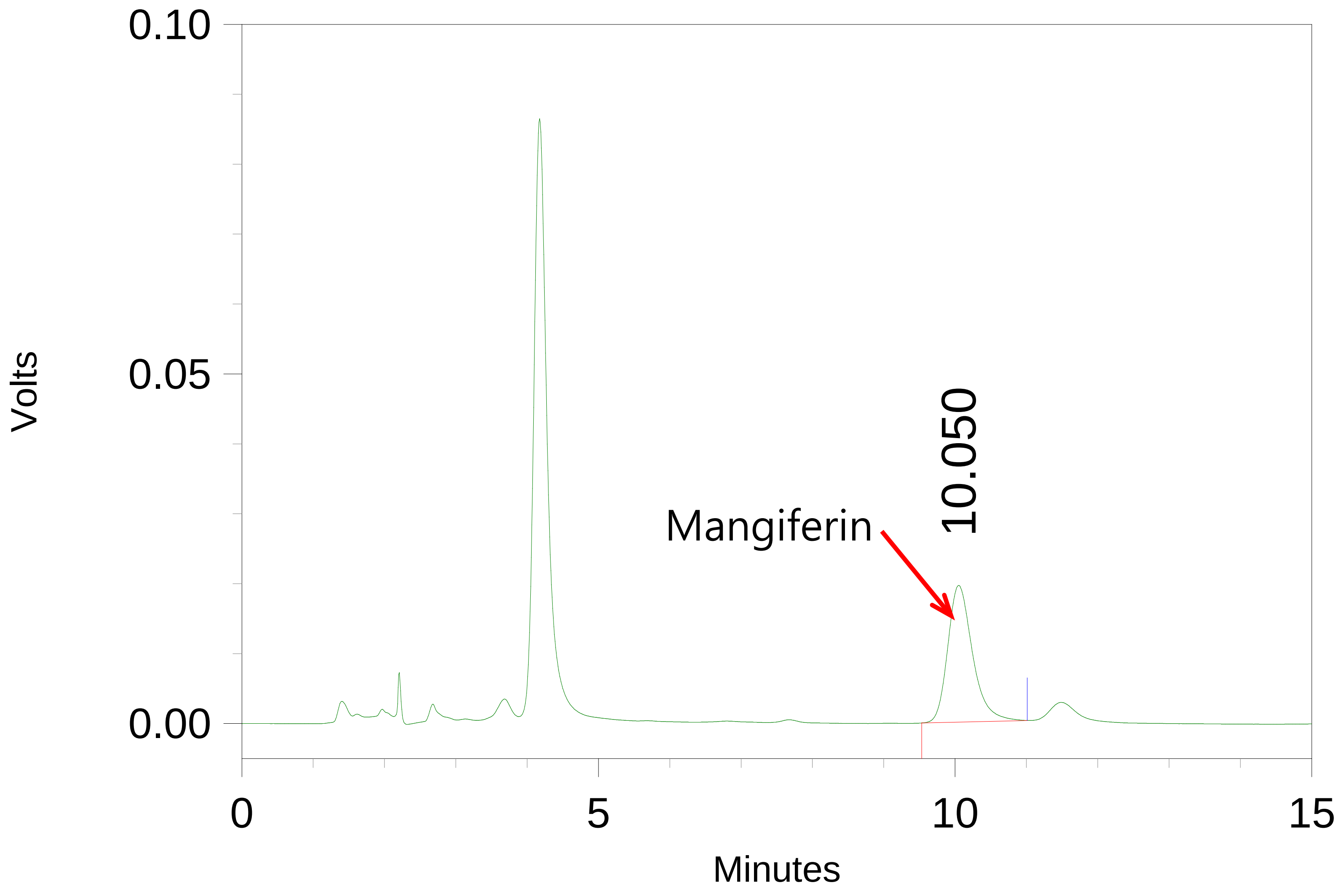

A. rhizoma -Timosaponin B II (HPLC-ELSD)

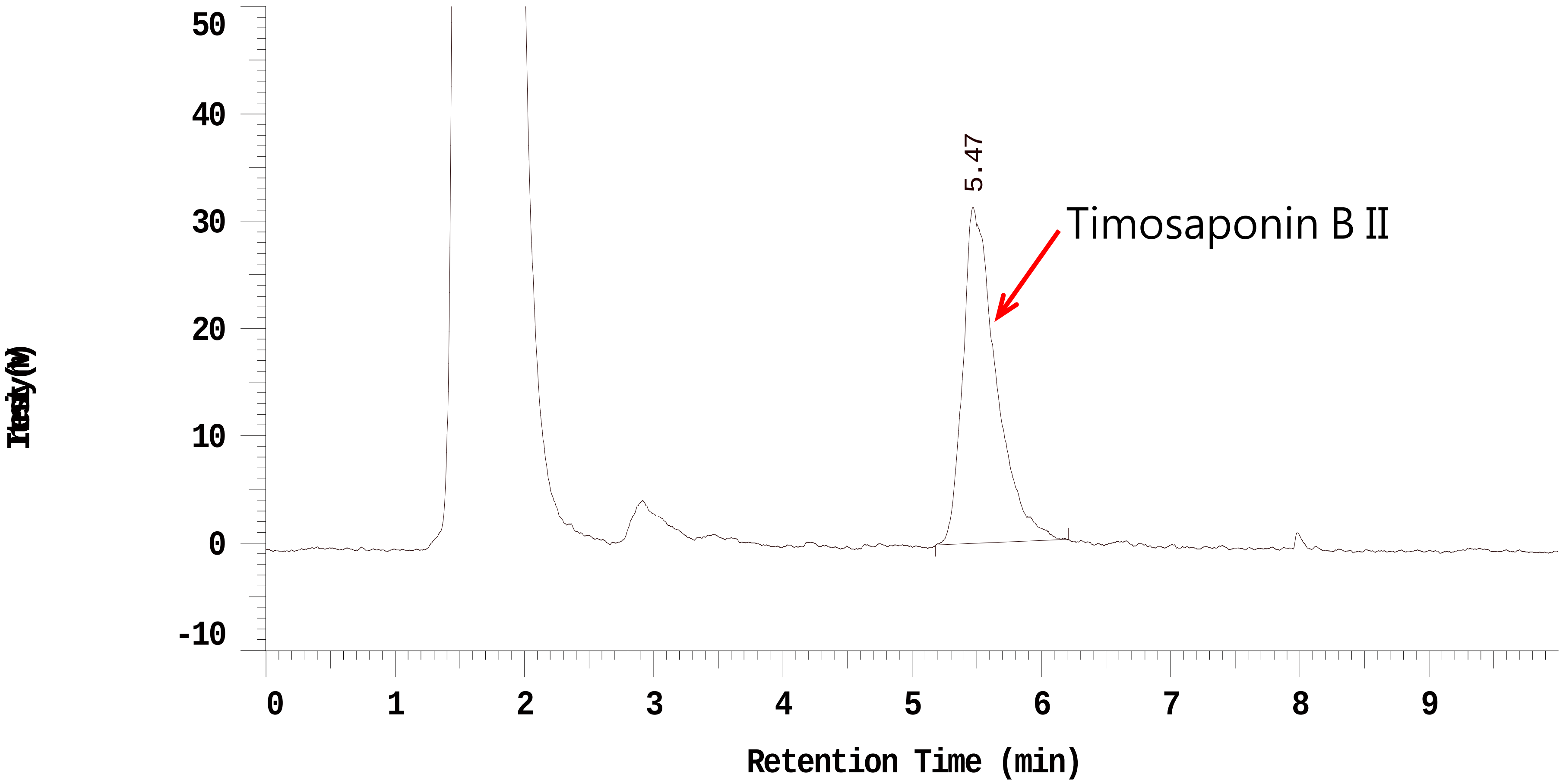

A. membranaceus -Astragaloside IV (LC-MS/MS)

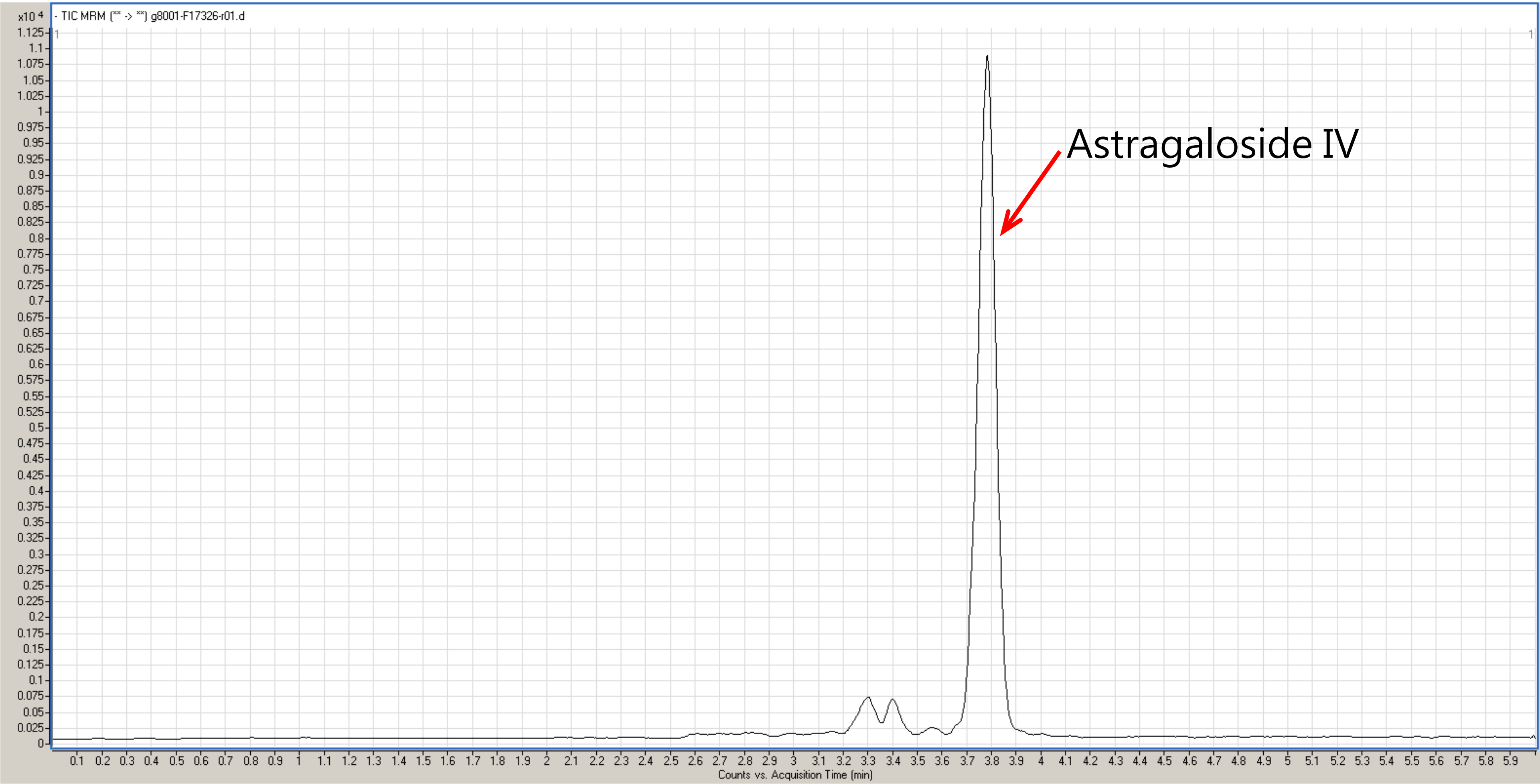

Supplement: Additional file 1: — High performance of liquid chromatography for Chinese herbal extracts. (PDF 310 kb) [file 12906_2017_1909_MOESM1_ESM.pdf]
